# Validation of the Spanish version of mackey childbirth satisfaction rating scale

**DOI:** 10.1186/s12884-016-0862-7

**Published:** 2016-04-16

**Authors:** Pablo Caballero, Beatriz E. Delgado-García, Isabel Orts-Cortes, Joaquin Moncho, Pamela Pereyra-Zamora, Andreu Nolasco

**Affiliations:** Department of Community Nursing, Preventive Medicine and Public Health and History of Science Health. Faculty of Health Sciences. University of Alicante, 03690 San Vicente del Raspeig, Alicante, Spain; Obstetrics and Gynecology Service, University Hospital of Vinalopó, Alicante, Spain; Department of Nursing, University of Alicante, Alicante, Spain

**Keywords:** Childbirth satisfaction, Labor, Survey research, Validation, Psychometric behavior, Reliability

## Abstract

**Background:**

The “Mackey Childbirth Satisfaction Rating Scale” (MCSRS) is a complete non-validated scale which includes the most important factors associated with maternal satisfaction. Our primary purpose was to describe the internal structure of the scale and validate the reliability and validity of concept of its Spanish version MCSRS-E.

**Methods:**

The MCSRS was translated into Spanish, back-translated and adapted to the Spanish population. It was then administered following a pilot test with women who met the study participant requirements. The scale structure was obtained by performing an exploratory factorial analysis using a sample of 304 women. The structures obtained were tested by conducting a confirmatory factorial analysis using a sample of 159 women. To test the validity of concept, the structure factors were correlated with expectations prior to childbirth experiences. McDonald’s omegas were calculated for each model to establish the reliability of each factor.

The study was carried out at four University Hospitals; Alicante, Elche, Torrevieja and Vinalopo Salud of Elche. The inclusion criteria were women aged 18–45 years old who had just delivered a singleton live baby at 38–42 weeks through vaginal delivery. Women who had difficulty speaking and understanding Spanish were excluded.

**Results:**

The process generated 5 different possible internal structures in a nested model more consistent with the theory than other internal structures of the MCSRS applied hitherto. All of them had good levels of validation and reliability.

**Conclusions:**

This nested model to explain internal structure of MCSRS-E can accommodate different clinical practice scenarios better than the other structures applied to date, and it is a flexible tool which can be used to identify the aspects that should be changed to improve maternal satisfaction and hence maternal health.

## Background

The outcomes of health care delivery are measured in terms of effectiveness and efficiency but also in terms of the individual’s experience as a patient. This experience involves pain, autonomy, a feeling of physical and mental well-being and satisfaction with the favorable results achieved [1], and provides a unique opportunity to better understand satisfaction with the quality of the health care provided [2]. Satisfaction with health care delivery is significantly associated with patients’ adherence to medical treatment [3], their quality of life [4] or simply improvements in their health status [5]. Therefore, patients’ experiences are increasingly being used internationally as an indicator of the quality and performance of health systems [6], and thousands of surveys are used by health care providers, administrators or policymakers to assess the quality of care, make decisions about provisions and organization of health care services, avoid malpractice and support a competitive edge in the health care area [7].

Patient satisfaction is a multi-dimensional concept that has received widespread research attention since the 1970s, and it has been evaluated from different points of view and with different goals [8], but without a measuring standard [9]. According to Weisman and Koch, patient satisfaction is only measurable by direct interaction. Consequently, many patient satisfaction surveys are designed specifically for each health service [10].

The field of obstetrics is not exempt from this trend. Maternal satisfaction (MS) after childbirth has consequences for the mother’s health and the well-being of her child [11], and a measure or index of MS provides a valuable outcome to improve the quality of maternity care services [12]. Although a low Apgar score, maternal or infant mortality rates and cesarean and instrumental delivery rates have been used to assess the outcomes of maternity care services, these are very restrictive parameters for assessing quality as they do not describe attitudes or processes [13–16]. In contrast, an assessment of MS with maternity care services makes it possible to determine the mother’s experience during childbirth and measure the quality of the care provided, because such care is centered on the patient’s needs [1, 12]. As a result, MS has become one of the most widely used indicators nowadays [11].

Various researchers have highlighted six main factors associated with MS besides demographic features; pain and relief, self-control, self-efficacy, expectations, partner support, and provision of opportunity to have immediate contact with the newborn [11, 12, 17–24].

Several questionnaires and scales have been created in multiple languages to measure the relation between MS and the childbirth experience [25], for instance the “Maternal Well-being in Childbirth Scale” [22], the “Women’s Views of Birth Labour Satisfaction Questionnaire” [17], the “Care in Obstetrics: Measure For Testing Satisfaction Scale” [23], the “Questionnaire Measuring Attitudes About Labor and Delivery” and the “Mackey Childbirth Satisfaction Rating Scale” (MCSRS) [11]. Of these, the latter (the MCSRS) is the most complete scale as it includes the most important factors associated with MS from our point of view. Created and used in the USA, the MCSRS has also been used in other countries such as the UK [26], Holland [27, 28], Belgium and Spain [29]. The MCSRS has 34 self-report items, all of which use a 5-point Likert scale from “very dissatisfied” to “very satisfied”, and it is aimed at mothers after a vaginal delivery. According to the authors, the MCSRS consists of six subscales: general satisfaction (three items), satisfaction with self (nine items), baby (three items), midwife (nine items), gynecologist (eight items), and partner (two items) [11]. Although all of the authors who have used the MCSRS have tested the internal consistency of the MCSRS and its subscales by means of Cronbach’s alpha, none of them have described or explored its factorial structure in depth, nor have they validated the psychometric behavior of its subscales. The only exploratory factorial analysis (EFA) of the MCSRS, which was carried out by Mas-Pons et al., concerned an adaptation to Spanish with two additional questions [29]. They used principal components analysis assuming continuous variables, but better statistical tools are now available that explicitly incorporate Likert scales [30]. In addition, no confirmatory factor analysis (CFA) has been performed on the subscales proposed by the authors of the MCSRS or the researchers who have used it. EFA and CFA analyses of the scale will contribute to a better use of the MCSRS and interpretation of the results of the subscales.

Defining the dimensions underlying the MCSRS and measuring the importance that women who have just delivered a baby give to each dimension would provide a better understanding of issues related to health care delivery, enabling practitioners to introduce changes that improve the experience of childbirth and thus improve the health of mothers and their newborns.

The primary goal of the present study was to describe the internal structure of the MCSRS by means of EFA and CFA, and to examine validity of concept and reliability.

## Methods

### Participants

Inclusion criteria were women aged 18–45 years old who had just delivered a singleton live baby at 38–42 weeks through vaginal delivery. Women who had difficulty speaking and understanding Spanish were excluded. For the CFA, women who had undergone an unplanned cesarean section were also included to expand the population to whom the questionnaire can be administered. Participants were informed of the nature of the study and assured that the confidentiality of their personal data would be maintained. All subjects gave their written informed consent.

The study sample was recruited at the Main University Hospital of Alicante, the Main University Hospital of Elche, the University Hospital of Torrevieja, and the University Hospital Vinalopó Salud of Elche. Together, these four hospitals covered the health needs of more than half a million people in 2010. The study was reviewed and approved by the ethics committees at the four participant hospitals.

### Measures

The MCSRS was translated and adapted in accordance with previous recommendations [31, 32]. It was translated into Spanish separately by two English-Spanish bilingual translators with a background in medical and health care texts and clinical experience. These two translations were used to reach an agreement resulting in the first translation into Spanish. Two other English-Spanish bilingual people, both native English speakers, translated this first translation back into English, after which a second version in Spanish was agreed upon. The definitive version was achieved after contrasting the opinions of four women who met the study participant requirements. The order and wording of the questions are shown in Table 1.

**Table 1:** Psychometrics factors by exploratory analysis factorial and theoretical model by Goodman et al. Rotated loading matrix to models from two to six factors. *N* = 304

Item	Question	2 F	RLM	3 F	RLM	4 F	RLM	5 F	RLM	6 F	RLM	T
Q1	Your overall labor experience	F1	0.623	F1	0,585	F5	0,715	F7	0.412	F7	0,383	T1
Q2	Your overall delivery experience	F1	0.569	F1	0,589	F5	0,563	F8	0.905	F8	0,952	T1
Q3	Your level of participation in decision-making during labor	F1	0.729	F1	0,664	F5	0,622	F7	0.364	F7	0,350	T2
Q4	Your level of participating in decision-making during delivery	F1	0.634	F1	0,637	F5	0,625	F8	0.544	F8	0,561	T2
Q5	Your ability to manage your labor contractions	F1	0.791	F1	0,789	F5	0,777	F7	0.772	F7	0,745	T2
Q6	Your level of comfort during labor	F1	0.638	F1	0,578	F5	0,633	F7	0.477	F7	0,450	T2
Q7	Your level of comfort during delivery	F1	0.635	F1	0,612	F5	0,564	F8	0.874	F8	0,919	T2
Q8	The control you had over your emotions during labor	F1	0.798	F1	0,845	F5	0,874	F7	0.801	F7	0,776	T2
Q9	The control you had over your emotions during delivery	F1	0.841	F1	0,891	F5	0,811	F8	0.660	F8	0,689	T2
Q10	The control you had over your actions during labor	F1	0.737	F1	0,779	F5	0,831	F7	0.909	F7	0,901	T2
Q11	The control you had over your actions during delivery	F1	0.858	F1	0,875	F5	0,758	F8	0.533	F8	0,548	T2
Q12	Your partner’s help and support during labor	F1	0.414	F1	0,421	F6	0,560	F6	0.624	F9	0,831	T6
Q13	Your partner’s help and support during delivery	F1	0.461	F1	0,448	F6	0,734	F6	0.755	F9	0,885	T6
Q14	Your baby’s physical condition at birth	F1	0,210	F1	0,236	F6	0,522	F6	0.487	F10	0,335	T3
Q15	The amount of time which passed before you first held your baby	F1	0.357	F1	0,354	F6	0,710	F6	0.654	F10	0,605	T3
Q16	The amount of time which passed before you first fed your baby	F1	0.378	F1	0,34	F6	0,593	F6	0.550	F10	0,728	T3
Q17	The physical care you received from the nursing staff during labor and delivery	F2	0.612	F3	0,882	F3	0,880	F3	0.862	F3	0,860	T4
Q18	The physical care you received from the medical staff during labor and delivery	F2	0.969	F4	0,738	F4	0,727	F4	0.727	F4	0,698	T5
Q19	The technical knowledge, ability, and competence of the nursing staff in labor and delivery	F2	0.670	F3	0,761	F3	0,724	F3	0.707	F3	0,696	T4
Q20	The technical knowledge, ability, and competence of the medical staff in labor and delivery	F2	0.876	F4	0,633	F4	0,621	F4	0.620	F4	0,631	T5
Q21	The amount of explanation or information received from the nursing staff in labor and delivery	F2	0.613	F3	0,845	F3	0,816	F3	0.826	F3	0,822	T4
Q22	The amount of explanation or information received from the medical staff in labor and delivery	F2	0.845	F4	0,654	F4	0,643	F4	0.646	F4	0,702	T5
Q23	The personal interest and attention given you by the nursing staff in labor and delivery	F2	0.775	F3	0,971	F3	0,933	F3	0.937	F3	0,935	T4
Q24	The personal interest and attention given you by the medical staff in labor and delivery	F2	0.958	F4	0,899	F4	0,884	F4	0.885	F4	0,864	T5
Q25	The help and support with breathing and relaxation which you received from the nursing staff in labor and delivery	F2	0.637	F3	0,726	F3	0,690	F3	0.718	F3	0,720	T4
Q26	The help and support with breathing and relaxation which you received from the medical staff in labor and delivery	F2	0.952	F4	0,912	F4	0,897	F4	0.904	F4	0,902	T5
Q27	The amount of time the nurses spent with you during labor	F2	0.737	F3	0,855	F3	0,855	F3	0.871	F3	0,873	T4
Q28	The amount of time the doctors spent with you during labor	F2	0.858	F4	0,89	F4	0,885	F4	0.896	F4	0,884	T5
Q29	The attitude of the nurses in labor and delivery	F2	0.620	F3	0,9	F3	0,931	F3	0.927	F3	0,924	T4
Q30	The attitude of the doctors in labor and delivery	F2	0.920	F4	1.101	F4	1,091	F4	1.096	F4	1,070	T5
Q31	The nursing staff’s sensitivity to your needs during labor and delivery	F2	0.656	F3	0,893	F3	0,872	F3	0.890	F3	0,893	T4
Q32	The medical staff’s sensitivity to your needs during labor and delivery	F2	0.995	F4	1.053	F4	1,038	F4	1.048	F4	1,051	T5
Q33	Overall, the care you received during labor and delivery	F2	0.703	F3	0,634	F3	0,611	F3	0.603	F3	0,601	T4
Q34	Overall, how satisfied or dissatisfied are you with your childbirth experience?	F1	0.419	F1	0,41	F5	0,443	F5	0.449	F5	0,480	T1

*XF:* model with *X* Factors, *RLM* Rotated Loading Matrix, *T* Theoretical Model proposed by Goodman et al. Theoretical structure T1: General satisfaction, T2: satisfaction with self, T3: baby, T4: midwife, T5: gynecologist, T6: partner. F1-F10 Names of factors obtained by EFA

Expectations prior to delivery were collected using a scale of 0–10 (where 0 means it did not fulfill my expectations at all, 5 means it was like I had imagined, and 10 means it was much better than I had expected).

### Procedure

The study sample was recruited in the obstetrics and gynecology patient rooms at the four participant hospitals from September 2010 to February 2011 by consecutive sample. Skilled health personnel, midwives, selected all women who met the requirements. At 12 h postpartum these were given a leaflet and told that it had instructions for completion, on one side, and the questionnaire, on the other. It was self-completed voluntarily within 36 h by women who had just delivered. The written informed consent and the leaflet were collected by the same personnel after 24 h.

### Analysis

The sample size selected for EFA was 10 subjects per item. This ratio, of 10:1, is recommended in the guide for validation and adaptation of an instrument [33]. A sample half this size was selected for CFA [34]. Consequently, the initial sample consisted of 510 women; 340 women for the EFA and 170 women for the CFA. Questionnaires that were not fully completed were excluded.

To determine the psychometric proprieties of the MCSRS, the software package FACTOR v9.20 was used to fit the EFAs [35, 36]. To test the appropriateness of applying a Factor Analysis, Bartlett’s sphericity test and the Kaiser-Meyer-Olkin index (KMO) were carried out. Multivariate skewness and kurtosis were measured to determine the multivariable normal distribution of the data by means of Mardia’s test.

FACTOR v9.20 used the polychoric correlation matrix to fit the models. The method used was weighted least squares and oblique rotation using Promin [37]. Five models were estimated, containing from 2 to 6 factors or dimensions. A parallel analysis based on 500 replications was conducted to suggest how many factors should be included to obtain the best model according to this analysis. To evaluate each model, different indices were calculated; the goodness of fit index (GFI), Bentler’s simplicity index (BSI), the loading simplicity index (LSI), which explains variance based on eigenvalues, and the root-mean-square residual (RMSR) [38]. The value of McDonald’s omega was calculated for each model to establish the reliability of each factor [38–40]. Lastly, correlations among factors were calculated to determine the inter-factor relation.

The second sample was used to validate the models obtained by EFA and the original model proposed by the authors, and a CFA was performed for each model via the R programming language and its “laavan” library [41]. Model fit in the second sample was measured by means of several indices and tests; in absolute terms, using the chi-square test, root-mean-square error of approximation (RMSEA), standardized-root-mean-square residual (SRMSR) and goodness of fit index (GFI); in weighted terms by the number of estimated parameters using the adjusted goodness of fit index (AGFI) and parsimonious goodness of fit index (PGFI); in comparison to the baseline model using the comparative fit index (CFI), Normed Fit Index (NFI) and non-normed fit index (NNFI), and taking into account both weighting by the number of estimated parameters and comparing it with the baseline model using the parsimony normed fit index (PNFI) [42]. In addition, the overall congruence index (OCI) and factor congruence index (FCI) were calculated for each model and factor to check congruencies between models calculated by EFA and the best possible models calculated in the second sample.

In the absence of a gold standard, we considered that expectations prior to delivery were strongly related to MS [11, 12, 18, 19]. Thus, to validate the concept, we assumed that any scale that measures or assesses MS must also be associated with expectations prior to delivery. Thus, we calculated the score in each factor and in the MCSRS for each woman and we measured the lineal relationship with expectations using the Pearson correlation coefficient [17, 18].

## Results

In the first 4 months, 390 women were recruited, 61 of whom did not take part in the study (15.6 %) and 15 did not complete all items (3.8 %); consequently, 304 participated in the EFA. In the following two months, 175 women were recruited, 16 of whom did not take part in the study (9.1 %) but all participants completed all items; thus, 159 participated in the CFA.

The average age of participants was 32.74 (SD ± 4.80). The predominant marital status was married (68.6 %). Planned pregnancies accounted for 71.8 % of cases, and 49 % of participants were primiparous. About half of the participants (53.9 %) had attended more than two sessions of maternal education. Nearly all women (96.8 %) had a main companion throughout most of the birthing process, and for most of them (95.4 %), this was their partner. In 65.7 % of cases, the onset of labor was spontaneous. Oxytocin was administered to 64.1 % of women at some point, and 79.1 % were attended by the same midwife throughout the birthing process. The most commonly used method of pharmacological pain relief was epidural analgesia (61.3 %). The average length of labor was 327.34 min (SD ± 217.54) and the average expulsive period was 53.86 min (SD ± 49.51).

The termination mode of delivery in the sample for AFE was: eutocic 86.2 % and instrumental delivery 13.8 %. The sample for the AFC distribution was: eutocic 67.8 %, instrumental delivery 14 %, and cesarean 18.2 %.

A high percentage (84.4 %) of newborns achieved a high Apgar score of 8 in the first minute. A similar percentage (83.5 %) of women had their first contact with the baby within the first 10 min of life, and contact lasted for the first two hours in 88.5 % of cases. In addition, 86.5 % of women also initiated breastfeeding within the first hour after delivery.

Bartlett’s sphericity test presented statistical significance (Statistic 7745.8, degree of freedom 561, *p* < 0.001) and the KMO index was 0.922, suggesting the need to apply an EFA. Mardia’s test showed statistical significance for multivariate kurtosis (Statistic 95.77, *p* < 0.001) although not for multivariate skewness; therefore, we could not assume a multivariate normal distribution and thus a principal component analysis was not applied. The parallel analysis suggested 2 or 4 factors. Table 1 shows the weights of each factor over the main item for models with 2–6 factors. The first factor in the models with 2 and 3 factors had loadings below the minimum required (0.3) in the 14^th^ item (“Your baby’s physical condition at birth”). The rest of the factors in models with 4, 5 and 6 factors had loadings above this minimum.

The indices used to assess model fit are given in Tables 2 and 3. A GFI value of 1 indicates a perfect fit, and all models obtained over 0.95 for this index. Kelly’s criterion is used to assess the RMSR, where values near to or lower than Kelly’s criterion can be considered excellent [35]. In this case, Kelly’s criterion was 0.057 with an RMSR value of 0.074 for the 2-factor model, which decreased to 0.033 for the 6-factor model. A BSI value equal to 1 indicates maximum simplicity, and thus BSI models with fewer factors will obtain higher values than models with many more factors. However, in this case the BSI decreased slightly, obtaining 0.995 for the 2-factor model and 0.967 for the 6-factor model. The LSII is used to compare different models with each other; however, the values ranged between 0.504 for the 6-factor model and 0.579 for the 4-factor model. The explained variance based on eigenvalues increased with the number of eigenvalues or of factors in the model. Hence, the explained variance increased from 0.557 to 0.738.

**Table 2:** Indices exploratory factorial analysis

Number of factors	2		3		4		5		6	
Goodness of Fit Index (GFI)	0,980		0,990		0,990		0,990		1,00	
Root Mean Square of Residuals (RMSR)	0,074		0,060		0,050		0,038		0,033	
Bentler’s simplicity index (BSI)	0,995		0,995		0,988		0,979		0,967	
Loading Simplicity Index (LSI)	0,561		0,572		0,579		0,533		0,504	
Explained Variance Based on eigenvalues	0,557		0,611		0,663		0,707		0,738	
Reliability	Factor		Factor		Factor		Factor		Factor	
Omega McDonal	1	0.973	1	0.932	1	0.928	1	0.898	1	0.896
	2	0.933	2	0.960	2	0.816	2	0.901	2	0.911
			3	0.971	3	0.959	3	0.816	3	0.865
					4	0.971	4	0.961	4	0.768
							5	0.972	5	0.961
									6	0.971

**Table 3:** Indices exploratory factorial analysis

Correlation between Factors. Models from 2 to 6 factors															
2 Factors		3 Factors		4 Factors			5 Factors				6 Factors				
	2	2	3	2	3	4	2	3	4	5	2	3	4	5	6
1	0.75	0.73	0.68	0.61	0.70	0.65	0.50	0.49	0.56	0.53	0.511	0.42	0.14	0.54	0.52
2			0.76		0.57	0.53		0.53	0.64	0.60		0.47	0.20	0.66	0.64
3						0.75			0.76	0.51			0.34	0.46	0.48
4										0.76				0.11	0.04
5															0.77

The oblique rotations yielded correlations between model factors; these correlations showed statistical significance for all models.

Table 4 gives the results of the CFA for the 2-factor and 6-factor models and for the original model proposed by the authors, as well as the reference levels used by the various indices calculated to indicate an excellent fit [42]. In terms of the null hypothesis, which stated that there was no difference between the original data and the fitted models, the chi-square test only yielded a significant result for the 2-factor model. Meanwhile, the RMSEA was zero for all models except the 2-factor model, for which it was 0.038, and the SRMSR reached the minimum for the 6-factor model (0.081) but increased to 0.131 for the 2-factor model, whereas it was 0.094 for the theoretical model. Consequently, all of them were above the reference level. None of the fitted models obtained a GFI above 0.95, but came close, from 0.894 for the 2-factor model to 0.949 for the 6-factor model, while the GFI was 0.930 for the theoretical model. Taking into account the number of parameters to estimate, all models obtained an AGFI and PGFI above 0.8 except the 2-factor model, which obtained a PGFI of 0.079. When the fitted models or theoretical model were compared with the baseline model, indices such as the CFI, NNFI and NFI showed a marked improvement, but when the number of estimated parameters was taken into account, only the 5-factor and 6-factor models obtained a PNFI above the reference level of 0.85. Regarding congruence, the models with 4, 5 and 6 factors obtained an OCI above the reference level of 0.85, but it was the 5-factor model which obtained the maximum value (0.919). However, 2 out of 6 factors in the theoretical model obtained a very low FCI; 0.385 and 0.289.

**Table 4:** Confirmatory Factorial Analysis

Number of factors	RL	2	3	4	5	6	T
Absolute Terms							
*X* ^2^ Chi-Square		643,4	463.4	440.0	317.9	311.2	423.5
(p-valor)	>0,05	0,000	0,973	0,996	1.000	1.000	0.998
Root Mean Square Error of Approximation (RMSEA)	<0,07	0.038	0.000	0.000	0.000	0.000	0.000
Standardized Root Mean Square Residual (SRMSR)	<0,08	0,131	0.102	0.096	0.090	0.081	0.094
Goodness of fit Index (GFI)	>0,95	0.894	0.924	0.928	0.938	0.949	0.930
Weighted by the number of estimated parameters							
Adjusted Goodness of Fit Index (AGFI)	>0,8	0.880	0.913	0.917	0.928	0.940	0.919
Parsimony Goodness of Fit Index (PGFI)	>0,8	0.790	0.813	0.812	0.815	0.816	0.800
Comparison to the baseline model							
Comparative Fit Index (CFI)	>0,95	0.973	1.000	1.000	1.000	1.000	1.000
Non-Normed Fit Index (NNFI)	>0,95	0,971	1.000	1.000	1.000	1.000	1.000
Normed Fit Index (NFI)	>0,90	0,868	0.905	0.910	0.920	0.936	0.913
Weighted by the number of estimated parameters and compared to the baseline model							
PNFI Parsimony Normed Fit Index	>0,85	0,814	0.845	0.845	0.850	0.860	0.833
Congruence Indices							
Factor							
1	>0,85	0.693	0.764	0.928	0.933	0.936	0.872
2	>0,85	0.723	0.826	0.883	0.920	0.926	0.926
3	>0,85		0.979	0.979	0.980	0.983	0.983
4	>0,85			0.514	0.843	0.716	0.716
5	>0,85				0.884	0.872	0.289
6	>0,85					0.333	0.385
OCI Overall Congruence Index	>0,85	0.705	0.822	0.860	0.919	0.877	0.815

*RL* Reference Level by Lomax and Schumacker, and Lorenzo-Seva to Congruence Indices

The EFA generated 5 models and 10 different factors. To check the validity of concept of these factors and the MCSRS, scores were correlated with expectations prior to delivery, and the results are shown in Table 5; all but one of these correlations were significant.

**Table 5:** Pearson correlation among prior expectations, factor scores and MCSRS score

Factor	F1	F2	F3	F4	F5	F6	F7	F8	F9	F10	MCSRS
Models which include the Factor	2 F 3 F	2 F	3 F 4 F 5 F 6 F	3 F 4 F 5 F 6 F	4 F	4 F 5 F	5 F 6 F	5 F 6 F	6 F	6 F	
Correlation	0,425	0,285	0,346	0,183	0,452	0,183	0,357	0,420	0,070	0,200	0.354
Significance (bilateral)	<0,001	0,001	<0,001	0,036	<0,001	0,037	<0,001	<0,001	0,428	0,020	<0,001

F1-F10 Names of factors obtained Factors by Exploratory Analysis Factorial. XF: Model with *X* Factors

## Discussion

The results of Bartlett’s sphericity test and KMO suggested a factorial analysis and Mardia’s test ruled out multivariate normality of the data. Furthermore, the Likert scale variables indicated the use of a polychoric matrix and weighted least squares method in the factorial analysis instead of a principal component analysis. Although the parallel analysis suggested 2-factor or 4-factor models, an in-depth analysis of indices from the EFA or CFA might yield more possible models and another interpretation.

The EFA showed that the GFI was almost 1 for all models, and thus they all fitted the data with sufficient accuracy. The RMSR provides an assessment of the differences between the data and the model fit, whereby a RMSR below 0.08 indicates a good fit [42]; in our study, all models obtained RMSRs lower than 0.08.

Since the addition of factors increases the quality of the fit, although not necessarily the quality of the model, the BSI penalizes an unjustified increase in factors. However, the results show a slight decrease in the BSI as the number of factors increased. Therefore, models with more factors could be taken into account. Similarly, the LSI, another index which measures the simplicity of a model, showed the same behavior as the BSI.

The variation explained by eigenvalues started from 55.7 % for the 2-factor model, indicating a fair model. Thus, from the point of view of the explained variation, the rest of the models would be better than the 2-factor model.

Cronbach’s alpha has long been widely used as an estimate of the reliability of a psychometric test, and the author of the MCSRS [11] and all other researchers who have used the scale have calculated the Cronbach’s alpha [27, 29]. However, Cronbach’s alpha has been heavily criticized as an indicator for measuring reliability [43, 44]. Consequently, McDonald’s omega should be calculated [40] rather than Cronbach’s alpha. All factors from all models obtained fair omegas, and therefore no factor can be rejected due to a lack of reliability. The test-retest proposed by Keszei et al. would provide a more accurate indication of reliability, but was not performed in this study [45].

The various indices and tests implemented in the CFA showed good results for all models in the second sample. The chi-square test is widely used to analyze model fit, although the evidence is not conclusive [42]. In this case, only the 2-factor model did not pass the test. However, the rest of the indices used to assess the quality of the fit in absolute terms (the RMSEA, SRMSR and GFI) and in relative terms (the AGFI and PGFI) indicated that all the models analyzed were satisfactory. Similarly, the OCI and FCI congruence indices showed good results for all factors and all models. Meanwhile, the theoretical model obtained similar results to the 2-factor model.

**Fig. 1:**
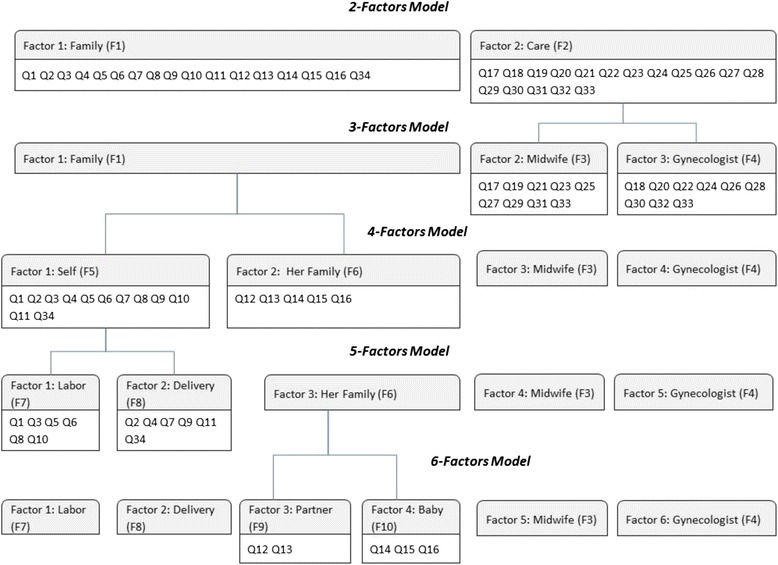
Nested model, from 2-Factors model to 6-factors model

In order to understand MS after childbirth using the fitted models, it is necessary to consider these models as a nested structure and to distinguish which items support each factor (Fig. 1). The results clearly show that if MS is explained by the 2-factor model, one of the factors could be named Family and the other one Care, where the Family factor would comprise the mother, the partner and the baby, while the Care factor would be obstetrical and gynecological care. The 3-factor model splits the Care factor into Midwife and Gynecologist, while the 4-factor model divides the Family factor into Self and Her Family. The 5-factor model might be the most interesting one; at this point, self-evaluation is chronologically divided into Labor and Delivery Period. Finally, the 6-factor model divides Her Family into the Baby and the Partner.

The original structure proposed by Goodman et al. and this nested model show some similarities and differences. The factors Midwife, Gynecologist, Baby and Partner appear in both models with the same supporting items. Nevertheless, the Overall factor does not exist in the nested model, and the original structure proposed by Goodman et al. does not distinguish between Labor and Delivery Period. We believe that Christiaens and Bracke (2009) might have obtained different results in their study entitled “Place of birth and satisfaction with childbirth in Belgium and the Netherlands” had they considered labor and delivery periods separately instead of as just one factor. In this case, they analyzed differences in MS between hospital birth (Belgium) and home birth (Netherlands). However, some of the women in the Dutch group spent the labor period at home but the delivery period at the hospital; consequently, the systems were not correctly compared and it was necessary to conduct another study to analyze this issue [28]. Regarding the Mas-Pons study (Mas-Pons R, et al. 2012), 2 more questions were added and an unsuitable statistical technique was employed, with the result that the structure of the psychometric factors obtained did not fit well in the theoretical framework of MS [29].

The most useful aspect of the nested model is that if the context of a study or the clinical setting does not differentiate between two factors, the model enables, for instance, a joint analysis of the care provided by the midwife and gynecologist, or maternal self-efficacy throughout the entire process.

Pearson’s correlation between expectations prior to delivery and the scores obtained by the factors and the MCSRS showed validity of concept for the MCSRS and all factors except one, the Baby factor, which only appears in the 6-factor model and did not present variability; 96.4 % of women obtained maximum scores. This result explains the lack of correlation.

Consequently, we conclude that the nested model proposed here yields a better and more in-depth description of MS within the theoretical framework of the MCSRS. In addition, it also allows us to identify naturally grouped factors and use this information to adapt them to the clinical setting.

### Limitations

Of the questionnaires administered, 15.6 % were not completed during the first 4 months. This may have affected the results and also indicates that participants may have found the MCSRS-E a long and difficult questionnaire to complete. However, new questionnaires have recently been reported, such as the Childbirth Experience Questionnaire (CEQ). This has been validated for use in Sweden by Dencker et al. 2010 [45] and in the UK by Walker et al. 2015 [46], and may present another alternative means to evaluate MS.

Although the wording of the questionnaire in the Spanish translation is acceptable in all Spanish-speaking countries, it is possible that the MCSRS-E may nevertheless require a cultural adaptation.

## Conclusion

The proposed nested model is in line with the theoretical framework. This structure can accommodate different clinical practice scenarios better than the other structures applied to date. Thus, if a particular clinical context requires that 2 or more factors be combined, this can only be achieved as indicated with the nested model if model validity is also to be maintained. However, MS is best understood by applying our clinical practice model with 6 factors, and we would recommend never using fewer than 4 factors. Therefore, this nested model is a flexible tool which can be used to identify the aspects that should be changed to improve MS and hence maternal health. In addition, the CFA inclusion criteria also encompassed women who underwent an unplanned cesarean section, extending the use of MCSRS.

## Abbreviations

MCSRS: mackey childbirth satisfaction rating scale
MCSRS-E: Spanish version of mackey childbirth satisfaction rating scale
MS: maternal satisfaction
EFA: exploratory factorial analysis
CFA: confirmatory factor analysis
KMO: kaiser-meyer-olkin index
GFI: goodness of fit index
BSI: bentler’s simplicity index
LSI: loading simplicity index
RMSR: root-mean-square residual
RMSEA: root-mean-square error of approximation
SRMSR: standardized-root-mean-square residual
AGFI: adjusted goodness of fit index
PGFI: parsimonious goodness of fit index
CFI: comparative fit index
NFI: normed fit index
NNFI: non-normed fit index
PNFI: parsimony normed fit index
OCI: overall congruence index
XF: model with X Factors
RLM: rotated loading matrix
T: theoretical model proposed by Goodman et al
RL: reference Level by lomax and schumacker, and lorenzo-seva to congruence indices
